# Comparison of the effect of a morton’s extension on plantar pressure distribution in female and male patients without deformities: A pre-post test study

**DOI:** 10.1371/journal.pone.0329949

**Published:** 2025-12-03

**Authors:** Anna Sánchez-Serena, Ricardo Becerro-de -Bengoa-Vallejo, Marta Elena Losa-Iglesias, Eduardo Pérez-Boal, Jorge Posada-Ordax, Eva María Martínez-Jiménez, Bibiana Trevissón-Redondo, Vicenta Martínez-Córcoles, Daniel López-López, Israel Casado-Hernández

**Affiliations:** 1 Department of Podiatry. Faculty of Health Sciences at Manresa. Universitat de Vic Universitat Central de Catalunya (UVic-UCC), Av. Universitària, 4-6, 08242 Manresa, Spain; 2 Departamento de Enfermería, Facultad de Enfermería Fisioterapia y Podología. Universidad Complutense de Madrid, Madrid, Spain; 3 Facultad de Ciencias de la Salud, Universidad Rey Juan Carlos, Alcorcón, Madrid, Spain; 4 Departamento de Enfermería, Facultad de Enfermería y Fisioterapia, Campus de Ponferrada, Universidad de León, León, Spain; 5 INSTIFEM.Instituto de Investigaciones feministas, Universidad Complutense de Madrid, Madrid, Spain; 6 Department of Behavioral Sciences and Health, Miguel Hernandez University of Elche, San Juan de Alicante, Spain; 7 Research, Health and Podiatry Group, Department of Health Sciences. Faculty of Nursing and Podiatry. Industrial Campus of Ferrol, Universidade da Coruña, Ferrol, Spain; Shahrood University of Technology, IRAN, ISLAMIC REPUBLIC OF

## Abstract

**Background:**

Plantar pressure measurement has become increasingly relevant in fields such as sports performance, rehabilitation, and podiatry. Its outcomes are influenced by factors including foot morphology, body weight, joint mobility, and biological sex, with documented anatomical and physiological differences between men and women potentially affecting load distribution. Morton’s extension, an orthotic device used to restore first ray function, has shown clinical efficacy in managing forefoot pathologies. However, limited research has examined its biomechanical effects with respect to sex. This study aimed to evaluate the differential impact of Morton’s extension on plantar pressure distribution in men and women, under both static and dynamic conditions.

**Methods:**

A sex comparing clinical trial intervention study was conducted with 18 men´s feet (38.11 ± 15.49 years) and 32 women´s feet (41.63 ± 15.22 years), who underwent plantar pressure assessments using a calibrated platform before and after the application of a Morton extension. Static and dynamic measurements were recorded under both conditions to evaluate changes in pressure distribution. A mixed models static analysis was done to check that there are differences by sex on foot.

**Findings:**

The following results summarize statistically significant differences observed between sexes under both static and dynamic conditions, with and without Morton’s extension. In static pressures without Morton’s extension, statistically significant results were found in P.Max.Rtp.(kPa) (p = 0.006) and Medium.pressure.Rtp.(kPa) (p < 0.001). With Morton’s extension in static conditions, significant differences were observed in P.Max.1M.(kPa) (p = 0.034), P.Max.Rtp.(kPa) (p = 0.017), and Medium.pressure.Rtp.(kPa) (p = 0.003). In dynamic pressures, before the intervention, statistically significant differences between sexes were observed in P.Max.1M.(kPa) (p = 0.023), Medium.pressure.1M.(kPa) (p = 0.008), Contact.Surfaces.(cm²) (p = 0.008), and Step.duration.(Milliseconds) (p = 0.048). After the intervention, additional significant differences were found in Contact.Surfaces.(cm²) (p = 0.009).

**Conclusion:**

The combined evidence indicates that Morton’s extension elicits clinically meaningful, sex-dependent alterations in plantar loading. By applying mixed-effects models we uncovered subtle intra- and interindividual patterns that conventional analyses may overlook. These results support sex-specific orthotic dosing (height/geometry) and argue for individualized prescription to maximize load redistribution, reduce focal overpressure and prevent site-specific pathology. Future work should validate optimal orthotic designs using dynamic 3D gait analysis and computational modelling.

## 1. Introduction

Plantar pressure measurement systems have gained significant attention in research, with key applications in sports performance analysis, injury prevention [1], ergonomic footwear design [2], and balance assessment [3]. In physiotherapy, they support rehabilitation programs [4], and medical diagnosis [5]. In podiatry, these systems are crucial for diabetes care, aiding in the prevention of foot ulcers [6] and are increasingly used in pediatric podiatry, such as in managing Sever’s disease in children [7].

With the aim of obtaining precise and reliable information, various plantar pressure measurement systems have been developed, among which pressure platforms hold a prominent place [8]. Baropodometry, in both its static and dynamic modalities, is considered the reference tool for analyzing plantar pressure distribution [9].

Evidence consistently shows that several factors, including anthropometric and kinematic determinants, influence plantar pressure distribution [10]. Body weight is a controversial factor; some studies find no correlation between body mass and plantar pressure [11] while others, such as Birtane and Tuna, associate obesity with a significant increase in plantar pressure values and contact area. Similarly, aging directly influences the distribution of plantar pressures [12,13]. From the age of 14 onwards, compared to earlier stages, alterations in this distribution have been observed according to sex. Interestingly, females tend to show an increase in plantar pressure in the toe and forefoot regions as age advances, compared to males. Joint range of motion is also considered a determining factor in plantar pressures. It has been described that limitations in joint mobility, such as reduced ankle dorsiflexion, can alter plantar pressure distribution by increasing pressure in the forefoot [14]. Regarding foot anatomy, navicular drop is associated with higher pressures in the hallux and medial forefoot, while navicular drift affects only the hallux [15]. Supinated feet show greater pressure in the lateral forefoot, while pronated feet exhibit increased pressure in the midfoot and medial forefoot [16]. In addition to these determinants, biological sex has also been identified as a potential factor influencing plantar pressure distribution. The influence of biological sex on plantar pressure distribution has been studied, but the results remain heterogeneous and sometimes contradictory, highlighting important gaps in current knowledge.

Men and women exhibit anatomical and physiological differences across various domains. In this context, forensic studies have demonstrated anthropometric variations in foot bone structure between the sexes [17], supporting the existence of sex-specific morphological traits. In line with this, Wunderlich et al. observed that female feet and legs display distinctive characteristics compared to male counterparts, particularly in the foot arch, lateral edge, first toe, and overall plantar surface [18]. Furthermore, Yamamoto et al. found increased plantar pressure in women, significantly greater in the big toe, other toes, forefoot, and medial foot region, both while standing and walking [19]. This functional difference may be partly explained by hormonal factors: women exhibit higher levels of relaxin and estradiol, which contribute to increased ligamentous laxity [20]. This laxity has been associated with a greater prevalence of flat feet [21], a condition that typically presents elevated plantar pressure in the medial arch; however, these differences in pressures have not been determined in dynamic conditions.Moreover, in the pathological domain, women show a greater propensity to develop forefoot disorders [22] and these musculoskeletal conditions and deformities have been associated with alterations in plantar pressure distribution [23]. Among these pathologies, hallux limitus stands out, as it also shows a higher prevalence in women [24].While the literature has associated this condition with alterations in plantar pressure distribution, no studies have directly compared these differences between sexes. Compared to healthy subjects, hallux limitus has been shown to cause a significant increase in peak pressure beneath the hallux and the first metatarsal head [25,26]. Additionally, in bilateral cases, an altered static plantar pressure distribution and greater contact area in the forefoot and heel have been reported [27].

Chung et al. indicated that men have higher peak pressure in the medial toe and all forefoot areas than women [28]. However, others, such as Murphy et al. [29], failed to confirm such differences, likely due to methodological limitations related to the segmentation of the foot and the restricted definition of the analyzed variables. In healthy patients, differences in plantar pressures between men and women have also been demonstrated.

On one hand, all these studies highlight the need to compare plantar pressures, as previously noted by other authors [30]. However, although there are studies suggesting sex-related differences in plantar pressures, these have not been conclusively confirmed. Despite this, the treatments applied to women’s feet, such as orthopedic treatment, are exactly the same as those applied to men’s feet. At present, there is no research examining the differences in the effects on plantar pressures or biomechanics between men and women.

To counteract the effects of excessive plantar pressure, foot orthoses have been identified as a highly effective tool for improving pressure distribution and reducing the incidence of lower-limb injuries [31]. In addition to enhancing comfort, orthoses have been shown to relieve forefoot pain in cases of rheumatoid arthritis, hallux abductus valgus, and secondary metatarsalgia by optimizing plantar pressure distribution [32]. Similarly, properly designed foot supports have been proven to reduce plantar pressure in patients at risk of ulceration [33]. Holmes et al. observed that a metatarsal bar effectively decreases pressure under the metatarsal heads [34].

Based on the studies conducted, there is limited evidence regardingthe effects of Morton’s extension on plantar pressure. In a previous study of ours, the application of Morton’s extension modified the pattern of plantar pressure distribution beneath the second and third metatarsal heads in healthy subjects, under both static and dynamic conditions [35], although potential sex-related differences in its effects were not analyzed. It is well established that adequate dorsiflexion of the first metatarsophalangeal joint during gait is essential for stabilizing the foot in the push-off phase and ensuring efficiency [36]. Morton’s extension is a device designed to restore the function of the first ray by helping to reduce the dorsiflexed position of the first metatarsal in the sagittal plane [37]. This therapeutic element is used in the management of conditions such as hallux limitus [31] and hallux rigidus [38], pathologies associated with incorrect positioning of the first ray axis in the sagittal plane. Additionally, other orthopedic elements, such as cut-out orthoses, have been investigated, with a recent study demonstrating a significant increase in the metatarsal angle declination, suggesting a relevant impact on foot biomechanics [39]. In parallel, within the field of biomedical research, there are studies that show significant differences in the response to pharmacological treatments between men and women. For example, the antithrombotic activity of cyclooxygenase (COX) inhibitors, a type of anti-inflammatory drug, has been found to produce more pronounced effects in men than in women [40]. Additionally, differences have been reported in recommended dosages and in the occurrence of side effects, with the latter being more frequent or intense in women [41].

Considering that sex is a variable that can influence biomechanical response [17–19,28] the present study investigated whether there are differences in the effect of Morton’s extension depending on the patient’s sex. Multiple studies have demonstrated that foot biomechanics differ between men and women, resulting in variations in plantar pressures and the prevalence of specific pathologies. Therefore, it is reasonable to affirm that women may present different plantar pressure patterns compared to men and may require distinct therapeutic approaches.

Studies analyzing the effects of Morton’s extension show a marked disparity in sex representation. Some research has been conducted exclusively on male populations [37], limiting the generalizability of the findings. Other studies have included both men and women, although with unequal distribution, with a predominance of female participants [35,42]. Conversely, certain investigations have involved a higher proportion of men than women [43]. This imbalance in sample composition, along with the absence of comparative studies evaluating the differential effects of plantar orthotic treatments between sexes, highlights the need for research incorporating a gender medicine perspective. Despite this gap, identical diagnostic and therapeutic criteria continue to be applied to both sexes, underscoring a lack of sex-specific clinical evidence.

Our hypothesis is that, due to the anthropometric and physiological characteristics of the female foot, plantar pressure distribution may be more homogeneous in women than in men, potentially resulting in greater therapeutic efficacy of Morton’s extension in the female population.

## 2. Materials and methods

### 2.1. Ethical considerations

This research received approval from the ethics committee of CEIC 20/235-E_TFM. Prior to the commencement of the study all participants provided their informed consent by signing the relevant form. The principles outlined in the Helsinki Declaration and all human experimentation regulations were duly adhered to [44]. The study was registered at ClinicalTrials.gov (NCT06715904 with initial release at 28/11/2024 initial recruitment at 28 February 25 and ends on 1 March 2025). The study was registered on ClinicalTrials.gov (identifier NCT06715904). The initial release was on November 28, 2024; recruitment began on February 28, 2025, and is expected to ends on 1 March 2025.

### 2.2. Participants

We computed the sample size necessary to achieve the study’s objective using the software G*Power® 3.1.9.7. We use a family Test with T-Student test of different means, with a priori calculation, with a big effect Size (D´Cohen’s = 0.90), Power of 0.80 and allocation ratio of 1.77 the sample needed in group 1 was 13 and the sample needed in group 2 is 23. This is based in other studies that comparing shape and antropometric sex differences that find significative differences in strength, forze and length with 39 healthy subjects (20 women 19 men) [45,46]. First group was determined to 13 subjects and second group for 23 subjects.

Considering the potential for attrition, 50 subjects were recruited for the study using a consecutive convenience sampling method. Among the healthy individuals attending the podiatry clinic for routine evaluations, those who met the inclusion criteria were consecutively enrolled.

The sample of 50 participants included 18 men´s feet and 32 women´s feet. The participants ages ranged from 18 to 60 years with a mean of 40.36 ± 15.26 years. The mean body mass index (BMI) was 24.68 ± 5.54 kg/m². There were no significant differences in age between the men and women, but significant differences were observed in weight, height, and BMI. Table 1 shows a detailed overview of the participants’ demographic and anthropometric characteristics.

**Table 1:** Demographics data for total population and sex.

Descriptive Data	Male (9) (n = 18 feet) Mean ± SD (CI95%)	Female (16) (n = 32 feet) Mean ± SD (CI95%)	Total (n = 50 feet) Mean ± SD (CI95%)	p-Value
**Age (years)**	38.11 ± 15.49 (30.96; 45.27)	41.63 ± 15.22 (36.35; 46.90)	40.36 ± 15.26 (31.13; 44.59)	0.440
**Height (cm)**	178.89 ± 4.19 (176.96; 180.82)	162.94 ± 5.35 (161.09; 164.79)	168.68 ± 9.16 (166.14; 171.22)	<0.001*
**Weight (kg)**	88.11 ± 14.97 (81.20; 95.03)	61.63 ± 12.46 (57.31; 65.94)	71.16 ± 18.46 (66.04; 76.28)	<0.001*
**BMI (kg/m²)**	27.52 ± 4.43 (25.48; 29.57)	23.08 ± 5.51 (21.17; 24.98)	24.68 ± 5.54 (23.14; 26.21)	0.005

Abbreviations: BMI body mass index; Kg kilograms; cm centimetres; SD standard deviation; CI: confidence interval. Student’s t-test for independent samples was applied. In all analyses. p < 0.05 (with a 95% confidence interval) was considered statistically significant.

The inclusion criteria were as follows: (1) no history of trauma to the foot; (2) the Silfverskiöld test was performed to assess ankle dorsiflexion with the knee extended and flexed at 90°, with the presence of at least 10° of dorsiflexion at the ankle required; (3) unrestricted motion of the functional subtalar joint of 30°; (4) unrestricted motion along the longitudinal axis of the midtarsal joint of 15°; (5) unrestricted non–weight-bearing motion of the first ray of at least 8 mm; (6) greater than 50° of dorsiflexion of the hallux to the first metatarsal bisection during non-weight-bearing (7) age greater than 18 years and less than 60 years; (8) no lower limb dysfunction or chronic injury at the time of data collection, nor any musculoskeletal or neurological condition that could interfere with gait or plantar pressure assessment (9) no evidence of a non-fixed deformity at the first metatarsophalangeal (MTP) joint and first metatarsal cuneiform joint. The exclusion criteria were: (1) plantar corns and calluses, (2) hallux valgus and lesser toe deformities, (3) diabetes, and (4) lower limb dysfunction. All participants complied with the eligibility criteria and signed the informed consent before any intervention.

### 2.3. Reliability of instruments

**Fig 1:**
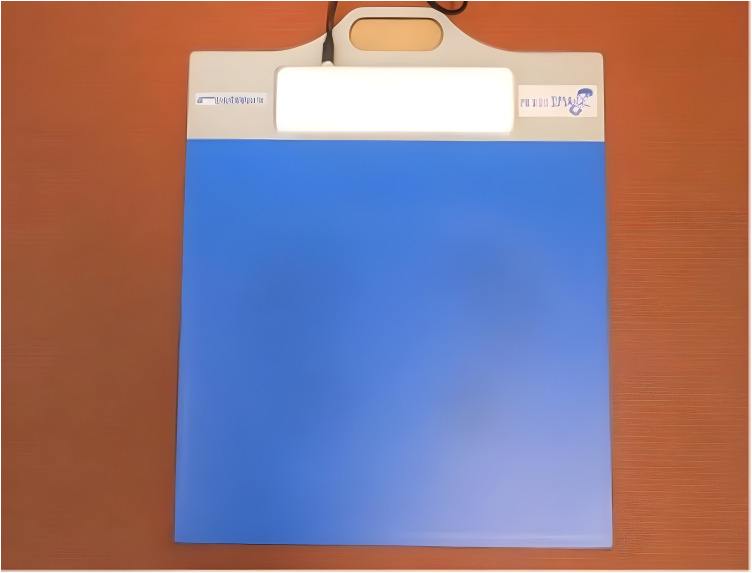
Footwork® pressure platform.

A Footwork® pressure platform (AM3-IST®, France) was used to assess plantar pressure distribution. The device featured a 400 × 400 mm active area, overall dimensions of 575 × 450 mm, a plate thickness of 4 mm, a pressure range of 10–1200 kPa, and a sampling frequency of 300 Hz.The platform consisted of 6,024 calibrated capacitive sensors (Fig 1). Following the manufacturer’s guidance, the equipment was individually calibrated before each test session. The Footwork® platform is a portable digital pressure-measurement system with capacitive sensors that was designed for clinical applications and has been used in various studies. This instrument has been shown to be highly reliable under both static and dynamic conditions [47].

Intra- and intersession reliability analyses have indicated substantial to almost perfect intraclass correlation coefficients (ICCs), along with low coefficients of variation, low standard error of measurement, and minimal percentage error. Intrasession ICCs ranged from 0.724 to 0.993 for static condition assessments and from 0.639 to 0.986 for dynamic assessments. Intersession ICCs ranged from 0.850 to 0.987 for static conditions and from 0.781 to 0.996 for dynamic conditions. In a similar study using the same pressure platform, reliability analysis indicated ICC values above 0.80 for the majority of variables assessed under both static and dynamic conditions [35]. The study was conducted in a comparable, non-pathological population in which plantar pressure parameters were assessed under both static and dynamic conditions. These findings confirm the device’s high consistency and suitability for clinical and research use.

### 2.4. Testing procedure

Static foot pressure was assessed with the subject in a standing position, with the tips of both feet aligned with the vertical and horizontal lines on the pressure platform.

Participants were instructed to maintain an upright posture, barefoot and without socks, looking forward, ensuring a stable position on the platform for 25 seconds [48]. The procedure was performed in three consecutive trials [49], and the mean value of these trials was used for analysis.

The dynamic foot pressure was assessed using the approach proposed by Palisanol et al. [50]. Participants started from a designated initial position, marked by the moment when the fifth step activated the pressure platform. They walked barefoot at a self-selected normal pace [51], ensuring that the entire length of the foot made contact with the platform during each trial to guarantee data accuracy.

**Fig 2:**
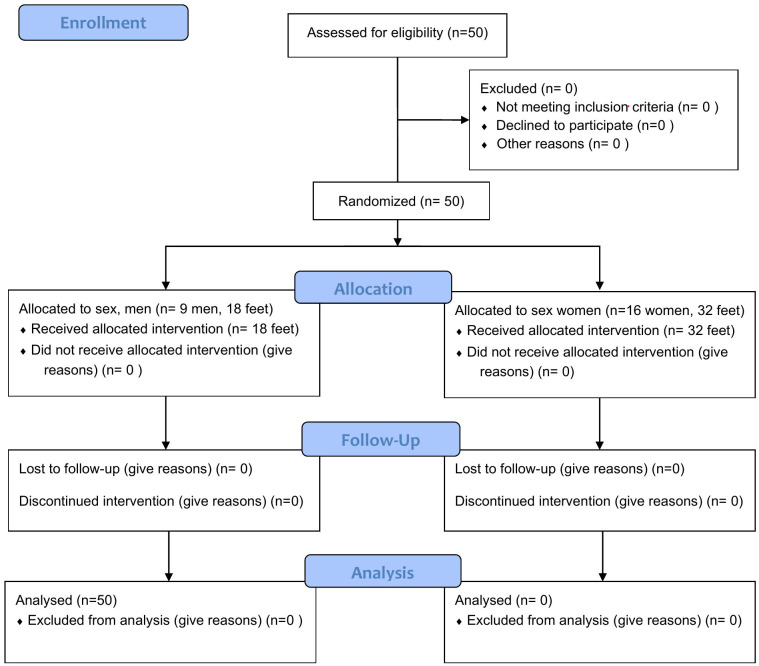
Consort Flow diagram.

To ensure participant familiarization with the testing procedure, two preliminary trials were conducted for each lower limb. Adequate recovery time was provided between protocols to prevent fatigue and guarantee optimal performance. Subsequently, three valid trials were recorded for each leg in an alternating sequence [49]. The first author randomly assigned the foot order for registration. A consistent protocol was administered by the same specialist for all study participants to uniformly affix. All participants were evaluated under two experimental conditions: (1) without the Morton’s extension, and (2) with a Morton’s extension made of 5-mm ethylene-vinyl acetate (EVA), positioned from the head of the first metatarsal to the proximal phalanx of the hallux [37], and secured with paper tape. During the procedure, it was verified that the Morton’s extension remained securely in place, ensuring a stable and consistent position throughout all measurements. Likewise, the clinician in charge confirmed for each trial that both the tape and the extension were correctly positioned [52]. In Fig 2 it could be seen the CONSORT flow diagram of research, where it can be seen that 50 feet of participants enrolled in the study, where no losses have occurred.

To systematically quantify plantar pressure distribution, two primary parameters were analyzed in specific anatomical regions. The first was the Medium plantar pressure (Medium.pressure), which is defined as the medium distribution of body weight across the plantar surface. The second parameter was the maximum plantar pressure (P.max), was defined as the peak pressure value recorded within each region of interest during a valid trial.

The variables are expressed as a combination of the abbreviation of the evaluated parameter (Medium.pressure or P.max) and the corresponding anatomical region according to the following scheme: P.Max.1M (kPa), maximum pressure at the head of the first metatarsal; P.Max.2M (kPa), maximum pressure at the head of the second metatarsal; P.Max.3M (kPa), maximum pressure at the head of the third metatarsal; P.Max.4M (kPa), maximum pressure at the head of the fourth metatarsal; P.Max.5M (kPa), maximum pressure at the head of the fifth metatarsal; Medium.pressure.1M (kPa), medium pressure at the head of the first metatarsal; Medium.pressure.2M (kPa), medium pressure at the head of the second metatarsal; Medium.pressure.3M (kPa), medium pressure at the head of the third metatarsal; Medium.pressure.4M (kPa), medium pressure at the head of the fourth metatarsal; Medium.pressure.5M (kPa), medium pressure at the head of the fifth metatarsal; P.Max.Mdf (kPa), maximum pressure in the midfoot; Medium.pressure.Mdf (kPa), medium pressure in the midfoot; P.Max.Rtp (kPa), maximum pressure in the rearfoot; Medium.pressure.Rtp (kPa), medium pressure in the rearfoot; P.Max.Hallux (kPa), maximum pressure in the hallux; Medium.pressure.Hallux (kPa), and medium pressure in the hallux.

After collecting the plantar pressure data, to enable precise measurements at specific anatomical locations, the foot was segmented longitudinally into the rearfoot, midfoot, and forefoot. The forefoot was further subdivided transversely into six regions corresponding to the first through fifth metatarsal heads (designated as 1M, 2M, 3M, 4M, and 5M), as well as the hallux. The foot consists of five metatarsal bones. The second through fifth metatarsals are referred to as the lesser metatarsals in order to distinguish them from the first metatarsal [53,54]. This segmentation has been performed previously by other authors and was carried out using the pressure platform software, which allowed for a detailed assessment of the pressure distribution across each of these anatomical zones [55].

### 2.5. Statistical analysis

All the data underwent normality testing using the Shapiro–Wilk test. The data were deemed to follow a normal distribution in cases of *p* > 0.05. The results of descriptive statistical analyses are presented as the mean ± standard deviation and a 95% confidence interval. An independent *t*-*t*est or Mann–Whitney U test was employed to compare variables between the sexes and before and after the treatment.

We performed an independent groups comparisons of women and men to observe the changes in each sex with the orthopedic treatment. An independent-samples t-test was used for variables with a normal distribution, and a Wilcoxon test was used for variables without a normal distribution. To verify statistically significant differences between sexes without weight implication in plantar pressure variables, we normalized the variables in relation to the weight of the subject. Subsequently, comparisons by sex with and without Morton’s extension were performed with an independent-sample *t*-test or Mann–Whitney U test. To determine how large a difference was, we calculated Cohen’s D, as shown in Tables 2–5. A value of 0.2 represents a small effect size, 0.5 represents a medium effect size, and 0.8 represents a large effect size.

**Table 2:** Comparison of plantar pressure variables by sex during static stance before and after.

Static Mode without Morton´s Extension						Static Mode with Morton´s Extension				
VARIABLE	Male (n = 18) Mean ± SD (IC95%)	Female (n = 32) Mean± SD (IC95%)	p-Value	Cohen´s D of non normalized variables comparison	p-Value from normalized variables by weight	Male (n = 18) Mean ± SD (IC95%)	Female (n = 32) Mean ± SD (IC95%)	p-Value	Cohen´s D of non normalized variables comparison	p-Value from normalized variables by weight
P.Max.1M. (kPa)	88.63 ± 41.40 (69.51;107.76)	93.88 ± 45.44 (78.14;109.63)	1.000^e^	0.119 ^d^	<0.001 ^d^ *	171.94 ± 67.06 (140.97; 202.92)	162.15 ± 52.05 (144.12; 180.18)	0.034* ^e^	0.858 ^d^	0.005 ^d^ *
P.Max.2M. (kPa)	125.69 ± 46.41 (104.25;147.13)	122.49 ± 44.64 (107.03;137.96)	1.000^e^	0.071 ^c^	<0.001 ^c^ *	78.19 ± 35.97 (61.57; 94.81)	80.33 ± 34.42 (68. 40; 92.25)	0.070 ^e^	−0.061 ^c^	0.002 ^c^
P.Max.3M. (kPa)	168.96 ± 45.51 (147.94; 189.99)	129.95 ± 46.34 (113.89; 146.00)	0.490 ^e^	0.847 ^c^	0.308 ^c^	104.45 ± 46.37 (83.02; 125.87)	79.73 ± 40.49 (65.70; 93.76)	0.984 ^e^	0.579 ^c^	0.542 ^c^
P.Max.4M. (kPa)	107.05 ± 34.21 (91.25; 122.86)	91.32 ± 46.72 (75.14; 107.51)	1.000 ^e^	0.368 ^d^	0.671 ^d^	91.83 ± 45.08 (71.01; 112.66)	69.29 ± 39.05 (55.76; 82.82)	1.000 ^e^	0.546 ^c^	0.540 ^c^
P.Max.5M. (kPa)	95.07 ± 66.76 (64.23; 125.92)	62.95 ± 46.89 (46.70; 79.20)	0.998 ^e^	0.587 ^d^	0.258 ^d^	93.93 ± 59.20 (66.58; 121.28)	66.78 ± 45.27 (51.10; 82.47)	0.830 ^e^	0.536 ^c^	0.803 ^c^
Medium pressure.1M.(kPa)	68.01 ± 32.89 (52.82; 83.21)	65.96 ± 34.75 (53.92; 78.00)	0.270 ^e^	0.060 ^d^	0.063 ^d^	139.04 ± 60.26 (111.20; 166.88)	119.99 ± 47.43 (103.56; 136.42)	0.570 ^e^	0.364 ^c^	0.053 ^c^
Medium pressure.2M.(kPa)	109.44 ± 42.53 (89.80; 129.09)	97.89 ± 37.13 (85.03; 110.76)	1.000 ^e^	0.295 ^c^	0.009 ^c^	63.02 ± 34.18 (47.23; 78.81)	64.37 ± 32.10 (53.24; 75.49)	1.000 ^e^	−0.041 ^d^	0.007 ^d^ *
Medium pressure.3M.(kPa)	145.27 ± 46.53 (123.78; 166.77)	104.36 ± 37.10 (91.51; 117.22)	0.940 ^e^	1.005 ^c^	0.867 ^c^	89.88 ± 44.76 (69.21; 110.56)	65.52 ± 37.86 (52.40; 78.63)	0.840 ^e^	0.603 ^d^	0.762 ^d^
Medium pressure.4M.(kPa)	89.96 ± 32.92 (74.75; 105.17)	65.23 ± 35.63 (52.89; 77.58)	1.000 ^e^	0.713 ^d^	0.671 ^d^	76.24 ± 39.25 (58.11; 94.38)	54.41 ± 34.98 (42.29; 66.53)	0.802 ^e^	0.597 ^d^	0.599 ^d^
Medium pressure.5M.(kPa)	71.80 ± 55.89 (45.98; 97.62)	39.56 ± 37.56 (26.54; 52.57)	0.822 ^e^	0.718 ^d^	0.258 ^d^	66.09 ± 49.34 (43.30; 88.88)	46.36 ± 37.49 (33.38; 33.38)	1.000 ^e^	0.469 ^d^	0.824 ^d^
P.Max.Mdf. (kPa)	32.34 ± 27.86 (19.47; 45.20)	10.56 ± 12.27 (6.31; 14.81)	1.000 ^e^	1.129^d d^	0.371 ^d^	24.77 ± 20.23 (15.42; 34.11)	12.45 ± 15.35 (7.13; 17.77)	1.000 ^e^	0.714 ^d^	0,044 ^d^ *
Medium. pressure.Mdf.(kPa)	20.32 ± 16.89 (12.52; 28.12)	6.04 ± 9.97 (2.58; 9.49)	1.000 ^e^	1.111 ^d^	0.005 ^d^ *	17.49 ± 16.67 (9.79; 25.19)	8.02 ± 12.32 (3.75; 12.29)	0.119 ^e^	0.676 ^d^	0.040 ^d^ *
P.Max.Rtp.(kPa)	252.72 ± 38.95 (234.72; 270.72)	230.30 ± 40.97 (216.11; 244.50)	0.006 ^e^	0.557 ^c^	<0.001^c^	246.07 ± 48.28 (223.77; 268.38)	213.27 ± 51.87 (195.30; 231.24)	0.017 ^e^	0.648 ^c^	0,006 ^c^
Medium.pressure.Rtp.(kPa)	222.99 ± 45.20 (202.11; 243.87)	198.78 ± 41.25 (184.49; 213.07)	≤0.001 ^e^	0.567 ^d^	0.002 ^d^	227.71 ± 45.00 (206.92; 248.50)	189.70 ± 46.58 (173.56; 205.84)	0.003 ^e^	0.826 ^c^	≤0,001 ^c^ *
P.Max.Hallux.(kPa)	24.86 ± 37.86 (7.37; 42.35)	793.99 ± 4363.53 (−717.87; 2305.85)	0.758 ^e^	−0.219 ^d^	0.078 ^d^	27.83 ± 51.50 (4.04; 51.62)	12.75 ± 19.24 (6.09; 19.42)	1.000 ^e^	0.439 ^d^	0.900 ^d^
Medium.pressure.Hallux.(kPa)	10.92 ± 18.49 (2.38; 19.47)	7.70 ± 8.57 (4.73; 10.67)	1.000 ^e^	0.249 ^d^	0,138^d^	16.14 ± 35.98 (−0.48; 32.76)	5.31 ± 9.14 (2.14; 8.48)	0.833 ^e^	0.478 ^d^	0,859 ^d^

Abbreviations: P. pressure; Max. Maximum; 1M. first metatarsal; 2 M. second metatarsal; 3M. third metatarsal; 4M. fourth metatarsal; 5M. fifth metatarsal; Mdf. midfoot; Rft. rearfoot; Kpa. kilopascals; SD. standard deviation. *p* < 0.05 (with a 95% confidence interval) was considered statistically significant. Cohen’s d. (^c^) T-Student or (^d^)U-Man Withney. (e)is a mixed models statics. *p*-value < 0.05 considered statistically significant.(*)

**Table 3:** Comparison of plantar pressure variables by sex during dynamic stance before and after.

VARIABLE	Dynamic Mode without Morton´s Extension					Dynamic Mode with Morton´s Extension				
	Male (n = 18) Mean ± SD (IC95%).	Female (n = 32) Mean± SD (IC95%)	p-Value	Cohen´s D of non normalized variables comparison	p-Value from normalizated variables by weight	Male (n = 18) Mean ± SD (IC95%).	Female (n = 32) Mean ± SD (IC95%).	p-Value	Cohen´s D of non normalized variables comparison	p-Value from normalizated variables by weight
P.Max.1M. (kPa)	294.67 ± 64.47 (264.88; 324.45)	257.01 ± 68.96 (233.12; 280.90)	0.023*^e^	0.202 ^d^	0,052^d^	323.11 ± 45.84 (301.94; 344.29)	347.54 ± 126.46 (303.72; 391.36)	0.079^e^	−0.445 ^c^	<0,001 ^c^ *
P.Max.2M. (kPa)	346.87 ± 26.49 (334.63; 359.11)	335.00 ± 36.73 (322.28; 347.73)	1.000	−0.029	<0,001 ^c^ *	272.82 ± 31.40 (258.32; 287.33)	268.06 ± 39.26 (254.46; 281.66)	0.938	0.178 ^c^	<0,001 ^c^ *
P.Max.3M. (kPa)	349.32 ± 35.45 (332.94; 365.69)	307.86 ± 39.56 (294.15; 321.57)	0.172	0.191 ^d^	<0,001 ^d^ *	328.35 ± 38.75 (310.45; 346.25)	296.14 ± 142.99 (246.60; 345.68)	0.398	0.375 ^c^	0,076 ^c^
P.Max.4M. (kPa)	284.51 ± 43.66 (264.34; 304.68)	241.59 ± 62.94 (219.79; 263.40)	1.000	−0.187	0.005 ^c^	286.17 ± 58.39 (259.20; 313.14)	233.30 ± 64.37 (210.99; 255.60)	0.839	−0.103 ^d^	0,032 ^d^
P.Max.5M. (kPa)	591.74 ± 1532.39 (−116.18; 1299.65)	174.78 ± 89.48 (143.78; 205.78)	0.740	0.236 ^d^	0.872 ^d^	277.13 ± 93.16 (234.09; 320.16)	192.35 ± 78.15 (165.27; 219.43)	0.343	0.370 ^d^	0,872 ^d^
Medium pressure.1M.(kPa)	140.81 ± 33.06 (125.54; 156.09)	104.12 ± 42.06 (89.54; 118.69)	0.008 ^e^ *	0.123	<0,001 ^c^ *	173.96 ± 23.26 (163.21; 184.70)	164.82 ± 36.93 (152.03; 177.61)	0.123 ^e^	0.299 ^c^	<0,001 ^c^ *
Medium pressure.2M.(kPa)	187.72 ± 32.03 (172.93; 202.52)	158.92 ± 26.35 (149.79; 168.05)	1.000 ^e^	0.082 ^d^	<0,001 ^d^ *	138.71 ± 32.70 (123.60; 153.81)	125.25 ± 32.40 (114.03; 136.48)	1.000 ^e^	0.286 ^c^	0,003 ^c^
Medium pressure.3M.(kPa)	192.65 ± 50.76 (169.20; 216.10)	149.69 ± 36.86 (136.92; 162.46)	1.000 ^e^	0.100	0,250 ^c^	170.20 ± 44.18 (149.79; 190.61)	122.15 ± 36.88 (109.37; 134.93)	1.000 ^e^	0.210 ^c^	0,725 ^c^
Medium pressure.4.M.(kPa)	127.14 ± 44.55 (106.56; 147.72)	103.67 ± 44.15 (88.37; 118.96)	1.000 ^e^	−0.187	0,185 ^c^	483.64 ± 1478.85 (−199.54; 1166.82)	106.55 ± 60.58 (85.56; 127.54)	0.908^e^	0.197 ^d^	0.599 ^d^
Medium pressure.5M.(kPa)	88.17 ± 50.84 (64.68;111.66)	61.16 ± 40.90 (46.99; 75.33)	0.740 ^e^	0.238 ^d^	0.258 ^d^	118.58 ± 54.49 (93.41; 143.75)	71.69 ± 49.95 (54.38; 89.00)	0.343^e^	0.068 ^d^	0,146 ^d^
P.Max.Mdf. (kPa)	48.88 ± 51.21 (25.22;72.53	24.96 ± 29.73 (14.66; 35.26)	1.000 ^e^	0.618 ^d^	0.371 ^d^	62.94 ± 58.73 (35.81; 90.07)	23.78 ± 27.88 (14.12; 33.45)	1.000 ^e^	0.943 ^d^	0.095 ^d^
Medium.pressure.Mdf. (kPa)	15.07 ± 15.66 (7.84; 22.31)	7.03 ± 9.68 (3.68; 10.38)	1.000 ^e^	0.662 ^d^	0.203 ^d^	17.44 ± 16.24 (9.94; 24.94)	6.77 ± 9.25 (3.57; 9.97)	0.330 ^e^	0.875 ^c^	0,037 ^c^ *
P.Max.Rft. (kPa)	345.77 ± 33.44 (330.32; 361.22)	322.96 ± 27.41 (313.47; 332.46)	1.000 ^e^	0.768	<0.00 1 ^c^ *	340.08 ± 27.19 (327.52; 352.64)	310.00 ± 34.61 (298.01; 321.99)	1.000 ^e^	0.935 ^c^	<0,001 ^c^ *
P. Medium pressure.Rft.(kPa)	145.81 ± 30.86 (131.56; 160.07)	131.39 ± 31.87 (120.35; 142.43)	1.000 ^e^	0.458	0,004 ^d^	135.61 ± 31.42 (121.10; 150.13)	132.46 ± 37.16 (119.59; 145.34)	0.998^e^	0.089 ^c^	<0,001 ^c^ *
P.Max.Hallux.(kPa)	242.81 ± 82.71 (204.60; 281.02)	259.62 ± 62.03 (238.13; 281.11)	0.330^e^	−0.240	<0.001 ^c^	218.31 ± 100.70 (171.79; 264.83)	214.64 ± 75.39 (188.52; 240.76)	0.849^e^	0.043 ^c^	0,007 ^c^ *
Medium pressure.Hallux.(kPa)	87.02 ± 41.03 (68.07; 105.97)	77.61 ± 36.57 (64.94; 90.28)	0.525^e^	0.246	<0,049 ^c^ *	66.12 ± 39.06 (48.08; 84.16)	57.92 ± 37.30 (44.99; 70.84)	0.255^e^	0.216	0,213 ^c^
Contact.Surfaces.(cm2)	121.28 ± 38.78 (103.36; 139.19)	94.65 ± 23.26 (86.59; 102.71)	0.008 ^c^ *	0.897	0,060 ^c^	115.92 ± 24.46 (104.62; 127.22)	94.35 ± 20.57 (87.22; 101.47)	0.009 ^c^ *	0.979	0,012 ^c^ *
Step.duration.(Milliseconds)	794.13 ± 131.93 (733.18; 855.08)	750.05 ± 119.36 (708.70; 791.41)	0.048*^e^	0.356	≤0.001 ^c^ *	799.81 ± 110.44 (748.80; 850.83)	736.21 ± 111.98 (697.41; 775.01)	1.000 ^e^	0.571 ^d^	≤0,001 ^d^ *

Abbreviations: P. pressure; Max. Maximum; 1M. first metatarsal; 2 M. second metatarsal; 3M. third metatarsal; 4M. fourth metatarsal; 5M. fifth metatarsal; Mdf. midfoot; Rft. rearfoot; Kpa. kilopascals; SD. standard deviation. *p* < 0.05 (with a 95% confidence interval) was considered statistically significant. Cohen’s d. (^c^) T-Student or (^d^)U-Man Withney. *p*-value < 0.05 considered statistically significant(*)

**Table 4:** Comparison of plantar pressure variables during static stance with and without Morton’s extension in males and females, including body weight–normalized values.

Static Mode without Morton´s Extension						Static Mode with Morton´s Extension				
VARIABLE	Male (n = 18) Mean ± SD (IC95%)	Female (n = 32) Mean± SD (IC95%)	p-Value	Cohen´s D of non normalized variables comparison	p-Value from normalized variables by weigth	Male (n = 18) Mean ± SD (IC95%)	Female (n = 32) Mean ± SD (IC95%)	p-Value	Cohen´s D of non normalized variables comparison	p-Value from normalized variables by weigth
P.Max.1M. (kPa)	88.63 ± 41.40 (69.51;107.76)	93.88 ± 45.44 (78.14;109.63)	0.688	0.119 ^d^	<0.001 ^d^ *	171.94 ± 67.06 (140.97; 202.92)	162.15 ± 52.05 (144.12; 180.18)	0.568	0.858 ^d^	0.005 ^d^ *
P.Max.2M. (kPa)	125.69 ± 46.41 (104.25;147.13)	122.49 ± 44.64 (107.03;137.96)	0.812	0.071 ^c^	<0.001 ^c^ *	78.19 ± 35.97 (61.57; 94.81)	80.33 ± 34.42 (68.40; 92.25)	0.836	−0.061 ^c^	0.002 ^c^
P.Max.3M. (kPa)	168.96 ± 45.51 (147.94; 189.99)	129.95 ± 46.34 (113.89; 146.00)	0.006*	0.847 ^c^	0.308 ^c^	104.45 ± 46.37 (83.02; 125.87)	79.73 ± 40.49 (65.70; 93.76)	0.055	0.579 ^c^	0.542 ^c^
P.Max.4M. (kPa)	107.05 ± 34.21 (91.25; 122.86)	91.32 ± 46.72 (75.14; 107.51)	0.217	0.368 ^d^	0.671 ^d^	91.83 ± 45.08 (71.01; 112.66)	69.29 ± 39.05 (55.76; 82.82)	0.070	0.546 ^c^	0.540 ^c^
P.Max.5M. (kPa)	95.07 ± 66.76 (64.23; 125.92)	62.95 ± 46.89 (46.70; 79.20)	0.052	0.587 ^d^	0.258 ^d^	93.93 ± 59.20 (66.58; 121.28)	66.78 ± 45.27 (51.10; 82.47)	0.075	0.536 ^c^	0.803 ^c^
Medium pressure.1M.(kPa)	68.01 ± 32.89 (52.82; 83.21)	65.96 ± 34.75 (53.92; 78.00)	0.839	0.060 ^d^	0.063 ^d^	139.04 ± 60.26 (111.20; 166.88)	119.99 ± 47.43 (103.56; 136.42)	0.223	0.364 ^c^	0.053 ^c^
Medium pressure.2M.(kPa)	109.44 ± 42.53 (89.80; 129.09)	97.89 ± 37.13 (85.03; 110.76)	0.321	0.295 ^c^	0.009 ^c^	63.02 ± 34.18 (47.23; 78.81)	64.37 ± 32.10 (53.24; 75.49)	0.890	−0.041 ^d^	0.007 ^d^ *
Medium pressure.3M.(kPa)	145.27 ± 46.53 (123.78; 166.77)	104.36 ± 37.10 (91.51; 117.22)	<0.001*	1.005 ^c^	0.867 ^c^	89.88 ± 44.76 (69.21; 110.56)	65.52 ± 37.86 (52.40; 78.63)	0.046 ^*^	0.603 ^d^	0.762 ^d^
Medium pressure.4M.(kPa)	89.96 ± 32.92 (74.75; 105.17)	65.23 ± 35.63 (52.89; 77.58)	0.019*	0.713 ^d^	0.671 ^d^	76.24 ± 39.25 (58.11; 94.38)	54.41 ± 34.98 (42.29; 66.53)	0.048 ^*^	0.597 ^d^	0.599 ^d^
Medium pressure.5M.(kPa)	71.80 ± 55.89 (45.98; 97.62)	39.56 ± 37.56 (26.54; 52.57)	0.019 ^*^	0.718 ^d^	0.258 ^d^	66.09 ± 49.34 (43.30; 88.88)	46.36 ± 37.49 (33.38; 33.38)	0.118	0.469 ^d^	0.824 ^d^
P.Max.Mdf. (kPa)	32.34 ± 27.86 (19.47; 45.20)	10.56 ± 12.27 (6.31; 14.81)	<0.001*	1.129^d d^	0.371 ^d^	24.77 ± 20.23 (15.42; 34.11)	12.45 ± 15.35 (7.13; 17.77)	0.019 ^*^	0.714 ^d^	0,044 ^d^ *
Medium. pressure.Mdf.(kPa)	20.32 ± 16.89 (12.52; 28.12)	6.04 ± 9.97 (2.58; 9.49)	<0.001*	1.111 ^d^	0.005 ^d^ *	17.49 ± 16.67 (9.79; 25.19)	8.02 ± 12.32 (3.75; 12.29)	0.026*	0.676 ^d^	0.040 ^d^ *
P.Max.Rtp.(kPa)	252.72 ± 38.95 (234.72; 270.72)	230.30 ± 40.97 (216.11; 244.50)	0.065	0.557 ^c^	<0.001^c^	246.07 ± 48.28 (223.77; 268.38)	213.27 ± 51.87 (195.30; 231.24)	0.033*	0.648 ^c^	0,006 ^c^
Medium.pressure.Rtp.(kPa)	222.99 ± 45.20 (202.11; 243.87)	198.78 ± 41.25 (184.49; 213.07)	0.060	0.567 ^d^	0.002 ^d^	227.71 ± 45.00 (206.92; 248.50)	189.70 ± 46.58 (173.56; 205.84)	0.007*	0.826 ^c^	≤0,001 ^c^ *
P.Max.Hallux.(kPa)	24.86 ± 37.86 (7.37; 42.35)	793.99 ± 4363.53 (−717.87; 2305.85)	0.460	−0.219 ^d^	0.078 ^d^	27.83 ± 51.50 (4.04; 51.62)	12.75 ± 19.24 (6.09; 19.42)	0.143	0.439 ^d^	0.900 ^d^
Medium.pressure.Hallux.(kPa)	10.92 ± 18.49 (2.38; 19.47)	7.70 ± 8.57 (4.73; 10.67)	0.403	0.249 ^d^	0,138^d^	16.14 ± 35.98 (−0.48; 32.76)	5.31 ± 9.14 (2.14; 8.48)	0.111	0.478 ^d^	0,859 ^d^

Abbreviations: P. pressure; Max. Maximum; 1M. first metatarsal; 2 M. second metatarsal; 3M. third metatarsal; 4M. fourth metatarsal; 5M. fifth metatarsal; Mdf. midfoot; Rft. rearfoot; Kpa. kilopascals; SD. standard deviation. *p* < 0.05 (with a 95% confidence interval) was considered statistically significant. Cohen’s d. (^c^) T-Student or (^d^)U-Man Withney. *p*-value < 0.05 considered statistically significant.(*)

**Table 5:** Comparison of plantar pressure variables during dynamic stance with and without Morton’s extension in males and females, including body weight–normalized values.

VARIABLE	Dynamic Mode without Morton´s Extension					Dynamic Mode with Morton´s Extension				
	Male (n = 18) Mean ± SD (IC95%).	Female (n = 32) Mean± SD (IC95%)	p-Value	Cohen´s D of non normalized variables comparison	p-Value from normalizated variables by weigth	Male (n = 18) Mean ± SD (IC95%).	Female (n = 32) Mean ± SD (IC95%).	p-Value	Cohen´s D of non normalized variables comparison	p-Value from normalizated variables by weigth
P.Max.1M. (kPa)	294.67 ± 64.47 (264.88; 324.45)	257.01 ± 68.96 (233.12; 280.90)	0.064 ^d^	0.202 ^d^	0,052^d^	323.11 ± 45.84 (301.94; 344.29)	347.54 ± 126.46 (303.72; 391.36)	0.435 ^c^	−0.445 ^c^	<0,001 ^c^ *
P.Max.2M. (kPa)	346.87 ± 26.49 (334.63; 359.11)	335.00 ± 36.73 (322.28; 347.73)	0.230 ^c^	−0.029	<0,001 ^c^ *	272.82 ± 31.40 (258.32; 287.33)	268.06 ± 39.26 (254.46; 281.66)	0.661 ^c^	0.178 ^c^	<0,001 ^c^ *
P.Max.3M. (kPa)	349.32 ± 35.45 (332.94; 365.69)	307.86 ± 39.56 (294.15; 321.57)	0.001 ^d^ *	0.191 ^d^	<0,001 ^d^ *	328.35 ± 38.75 (310.45; 346.25)	296.14 ± 142.99 (246.60; 345.68)	0.356 ^c^	0.375 ^c^	0,076 ^c^
P.Max.4M. (kPa)	284.51 ± 43.66 (264.34; 304.68)	241.59 ± 62.94 (219.79; 263.40)	0.014 ^c^	−0.187	0.005 ^c^	286.17 ± 58.39 (259.20; 313.14)	233.30 ± 64.37 (210.99; 255.60)	0.006*	−0.103 ^d^	0,032 ^d^
P.Max.5M. (kPa)	591.74 ± 1532.39 (−116.18; 1299.65)	174.78 ± 89.48 (143.78; 205.78)	0.128 ^d^	0.236 ^d^	0.872 ^d^	277.13 ± 93.16 (234.09; 320.16)	192.35 ± 78.15 (165.27; 219.43)	0.001*	0.370 ^d^	0,872 ^d^
Medium pressure.1M.(kPa)	140.81 ± 33.06 (125.54; 156.09)	104.12 ± 42.06 (89.54; 118.69)	0.003 ^c^ *	0.123	<0,001 ^c^ *	173.96 ± 23.26 (163.21; 184.70)	164.82 ± 36.93 (152.03; 177.61)	0.348 ^c^	0.299 ^c^	<0,001 ^c^ *
Medium pressure.2M.(kPa)	187.72 ± 32.03 (172.93; 202.52)	158.92 ± 26.35 (149.79; 168.05)	0.001 ^d^ *	0.082 ^d^	<0,001 ^d^ *	138.71 ± 32.70 (123.60; 153.81)	125.25 ± 32.40 (114.03; 136.48)	0.166 ^c^	0.286 ^c^	0,003 ^c^
Medium pressure.3M.(kPa)	192.65 ± 50.76 (169.20; 216.10)	149.69 ± 36.86 (136.92; 162.46)	0.001 ^c^ *	0.100	0,250 ^c^	170.20 ± 44.18 (149.79; 190.61)	122.15 ± 36.88 (109.37; 134.93)	<0.001 ^c^ *	0.210 ^c^	0,725 ^c^
Medium pressure.4.M.(kPa)	127.14 ± 44.55 (106.56; 147.72)	103.67 ± 44.15 (88.37; 118.96)	0.078 ^c^	−0.187	0,185 ^c^	483.64 ± 1478.85 (−199.54; 1166.82)	106.55 ± 60.58 (85.56; 127.54)	0.153 ^d^	0.197 ^d^	0.599 ^d^
Medium pressure.5M.(kPa)	88.17 ± 50.84 (64.68;111.66)	61.16 ± 40.90 (46.99; 75.33)	0.046 ^d *^	0.238 ^d^	0.258 ^d^	118.58 ± 54.49 (93.41; 143.75)	71.69 ± 49.95 (54.38; 89.00)	0.003 ^d^ *	0.068 ^d^	0,146 ^d^
P.Max.Mdf. (kPa)	48.88 ± 51.21 (25.22;72.53	24.96 ± 29.73 (14.66; 35.26)	0.041 ^d^ *	0.618 ^d^	0.371 ^d^	62.94 ± 58.73 (35.81; 90.07)	23.78 ± 27.88 (14.12; 33.45)	0.002 ^d^ *	0.943 ^d^	0.095 ^d^
Medium.pressure.Mdf. (kPa)	15.07 ± 15.66 (7.84; 22.31)	7.03 ± 9.68 (3.68; 10.38)	0.029 ^d^ *	0.662 ^d^	0.203 ^d^	17.44 ± 16.24 (9.94; 24.94)	6.77 ± 9.25 (3.57; 9.97)	0.005 ^c^ *	0.875 ^c^	0,037 ^c^ *
P.Max.Rft. (kPa)	345.77 ± 33.44 (330.32; 361.22)	322.96 ± 27.41 (313.47; 332.46)	0.012 ^c^ *	0.768	<0.00 1 ^c^ *	340.08 ± 27.19 (327.52; 352.64)	310.00 ± 34.61 (298.01; 321.99)	0.003 ^c^ *	0.935 ^c^	<0,001 ^c^ *
P. Medium pressure.Rft.(kPa)	145.81 ± 30.86 (131.56; 160.07)	131.39 ± 31.87 (120.35; 142.43)	0.127 ^d^	0.458	0,004 ^d^	135.61 ± 31.42 (121.10; 150.13)	132.46 ± 37.16 (119.59; 145.34)	0.763 ^c^	0.089 ^c^	<0,001 ^c^ *
P.Max.Hallux.(kPa)	242.81 ± 82.71 (204.60; 281.02)	259.62 ± 62.03 (238.13; 281.11)	0.420 ^c^	−0.240	<0.001 ^c^	218.31 ± 100.70 (171.79; 264.83)	214.64 ± 75.39 (188.52; 240.76)	0.884 ^c^	0.043 ^c^	0,007 ^c^ *
Medium pressure.Hallux.(kPa)	87.02 ± 41.03 (68.07; 105.97)	77.61 ± 36.57 (64.94; 90.28)	0.408 ^c^	0.246	<0,049 ^c^ *	66.12 ± 39.06 (48.08; 84.16)	57.92 ± 37.30 (44.99; 70.84)	0.466 ^c^	0.216	0,213 ^c^
Contact.Surfaces.(cm2)	121.28 ± 38.78 (103.36; 139.19)	94.65 ± 23.26 (86.59; 102.71)	0.004 ^c^ *	0.897	0,060 ^c^	115.92 ± 24.46 (104.62; 127.22)	94.35 ± 20.57 (87.22; 101.47)	0.002 ^c^ *	0.979	0,012 ^c^ *
Step.duration.(Milliseconds)	794.13 ± 131.93 (733.18; 855.08)	750.05 ± 119.36 (708.70; 791.41)	0.233 ^c^	0.356	≤0.001 ^c^ *	799.81 ± 110.44 (748.80; 850.83)	736.21 ± 111.98 (697.41; 775.01)	0.059 ^d^	0.571 ^d^	≤0,001 ^d^ *

Abbreviations: P. pressure; Max. Maximum; 1M. first metatarsal; 2 M. second metatarsal; 3M. third metatarsal; 4M. fourth metatarsal; 5M. fifth metatarsal; Mdf. midfoot; Rft. rearfoot; Kpa. kilopascals; SD. standard deviation. *p* < 0.05 (with a 95% confidence interval) was considered statistically significant. Cohen’s d. (^c^) T-Student or (^d^)U-Man Withney. *p*-value < 0.05 considered statistically significant(*)

All statistical analyses were conducted using SPSS 17.0 (SPSS Inc.. Chicago. IL. USA). The measurement data collected before and after the implementation of the Morton’s extension were processed while considering both static and dynamic aspects within a repeated-measures design. Statistical significance was established at a *p* value of <0.05, and 95% confidence intervals were used.

## 3. Results

**Table 2** presents a detailed summary of plantar pressure variables assessed under static conditions, stratified by sex and analyzed across two experimental conditions: before the intervention (without Morton’s extension) and after its application (with Morton’s extension). The table includes measurements of maximum and mean pressure at the five metatarsal heads (1M to 5M), the midfoot (Mdf), the rearfoot (Rtp), and the hallux. Before the intervention, statistically significant differences between sexes were observed in P.Max.Rtp (kPa) (p = 0.006), and Medium.pressure.Rtp (kPa) (p ≤ 0.001). After the intervention, additional significant differences were found in P.Max.1M (kPa) (p = 0.034) and Medium.pressure.Rtp (kPa) (p = 0.003).

**Table 3** presents a detailed summary of plantar pressure variables assessed under dynamic conditions, stratified by sex and analyzed across two experimental conditions: before the intervention (without Morton’s extension) and after its application (with Morton’s extension). The table includes measurements of maximum and mean pressure at the five metatarsal heads (1M to 5M), the midfoot (Mdf), the rearfoot (Rtp), and the hallux, as well as contact surface area and step duration, along with statistical comparisons between groups. Before the intervention, statistically significant differences between sexes were observed in P.Max.1M.(kPa) (p = 0.023), Medium pressure.1M.(kPa) (p = 0.008), Contact.Surfaces.(cm2) (p = 0.008) and Step.duration.(Milliseconds) (p = 0.048)After the intervention, additional significant differences were found in Contact.Surfaces.(cm2)) (p = 0.009).

**Fig 3:**
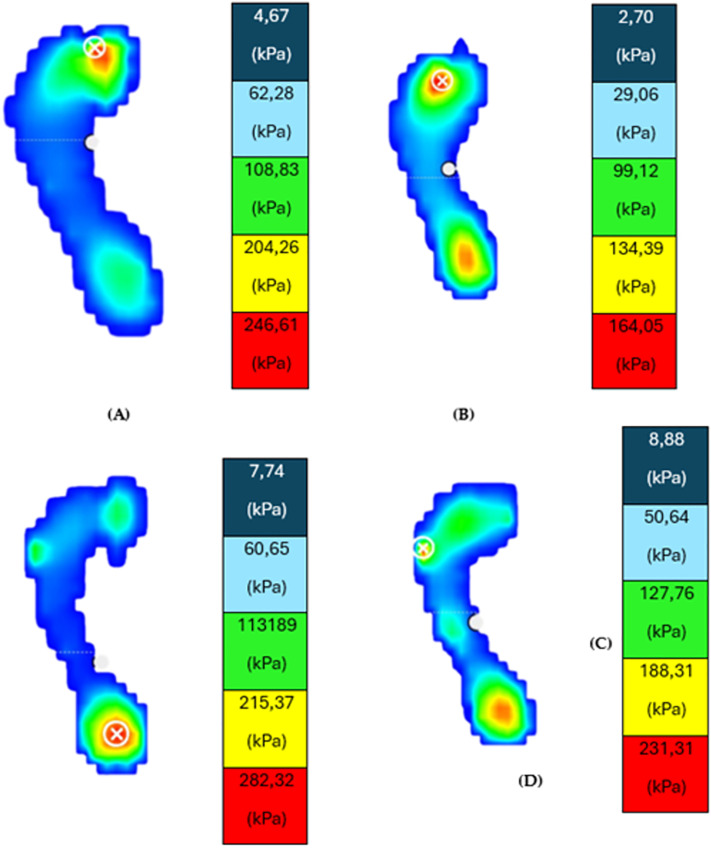
Representative image of plantar pressures obtained on the pressure platform from one participant under static conditions before and after the orthotic simulation.

Two parameters were evaluated in each analysis, static and dynamic. A Fig with a representative man and woman subjects before and after Morton Extension evaluation it is showed in Fig 3 under static condition. (See Fig 3)

**Fig 3A and 3B** display the static plantar pressure distribution in a female participant, comparing conditions **with** and **without** the application of a Morton’s extension. **Fig 3C and 3D** present the corresponding plantar pressure maps for a male participant under the same two conditions. The **circle with a cross** indicates the region of **maximum plantar pressure**. Pressure intensity is colour-coded from lowest to highest as follows: **dark blue**, **light blue**, **green**, **yellow**, and **red**, with red representing the **peak pressure zone**.

**Fig 4:**
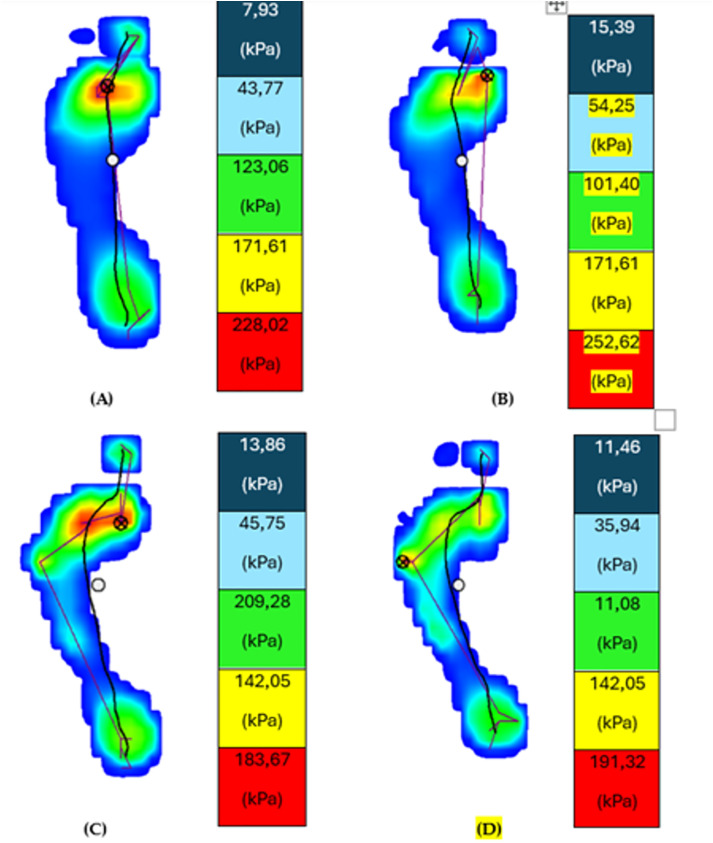
Representative image of plantar pressures obtained on the pressure platform from one participant under dynamic conditions before and after the orthotic simulation.

A Fig with a representative man and woman subjects before and after Morton Extension evaluation it is showed in Fig 4 under dynamic condition (See Fig 4).

**Fig 4A and 4B** display the **dynamic plantar pressure distribution** in a female participant, comparing conditions **with** and **without** the application of a Morton’s extension. **Fig 4C and 4D** present the corresponding dynamic plantar pressure maps for a male participant under the same two conditions. The **circle with a cross** indicates the region of **maximum plantar pressure**. Pressure intensity is colour-coded from lowest to highest as follows: **dark blue**, **light blue**, **green**, **yellow**, and **red**, with red representing the **peak pressure zone**.

Table 4 shows the results of statistical tests for static mode. Statistically significant differences in plantar pressure were observed between men and women without the Morton extension in P.Max.3M.(kPa), Medium.pressure.3M.(kPa), Medium.pressure.4M.(kPa), Medium.pressure 5M.(kPa), P.Max.Mdp.(kPa), and Medium.pressure.Mdp.(kPa). When values were normalized by weight, additional significant differences were observed in: P.Max.1M.(kPa), P.Max.2M.(kPa), Medium pressure.2M.(kPa), Medium pressure.Mdf.(kPa), P.Max.Rtp.(kPa) and Medium pressure.Rtp.(kPa). With the Morton extension, statistically significant differences were observed in Medium pressure..3M.(kPa). Medium pressure.4.M.(kPa). P.Max.Mdp.(kPa). Medium pressure.Mdp.(kPa). P.Max.Rtp.(kPa). Medium pressure..Rtp.(kPa). When values were normalized by weight, additional significant differences were observed in: P.Max.1M.(kPa), P.Max.2M.(kPa), Medium pressure.2M.(kPa), P.Max.Mdf.(kPa), Medium. pressure.Mdf.(kPa), Medium.pressure.Rtp.(kPa), P.Max.Rtp.(kPa), Medium.pressure.Rtp.(kPa)

Table 5 shows the results for dynamic mode. Statistically significant differences in plantar pressure were observed between men and women without the Morton extension in P.Max.3M.(kPa), Medium pressure.1M.(kPa), Medium pressure.2M.(kPa), Medium pressure.3M.(kPa), Medium pressure.5M.(kPa), P.Max.Mdf.(kPa), Medium pressure.Mdf.(kPa), P.Max.Rft.(kPa), and Contact.Surfaces.(cm²). When values were normalized by weight, additional significant differences were observed in: P.Max.1M.(kPa), P.Max.2M.(kPa), P.Max.3M.(kPa), Medium pressure.1M.(kPa), Medium pressure.2M.(kPa), P.Max.Rft.(kPa), P.Max.Hallux.(kPa), Medium pressure.Hallux.(kPa) and Step.duration.(Milliseconds). With the Morton extension, statistically significant differences were observed in P.Max.4M.(kPa), P.Max.5M.(kPa), Medium pressure.3M.(kPa), Medium pressure.5M.(kPa), P.Max.Mdf.(kPa), Medium pressure.Mdf.(kPa), P.Max.Rft.(kPa), and Contact.Surfaces.(cm²). When values were normalized by weight, additional significant differences were observed in: P.Max.1M.(kPa), P.Max.2M.(kPa), P.Max.4M.(kPa), Medium pressure.1M.(kPa), Medium pressure.2M.(kPa), Medium.pressure.Mdf.(kPa), P.Max.Rft.(kPa), P. Medium pressure.Rft.(kPa), P.Max.Hallux.(kPa), Contact.Surfaces.(cm2) and Step.duration.(Milliseconds).

**Fig 5:**
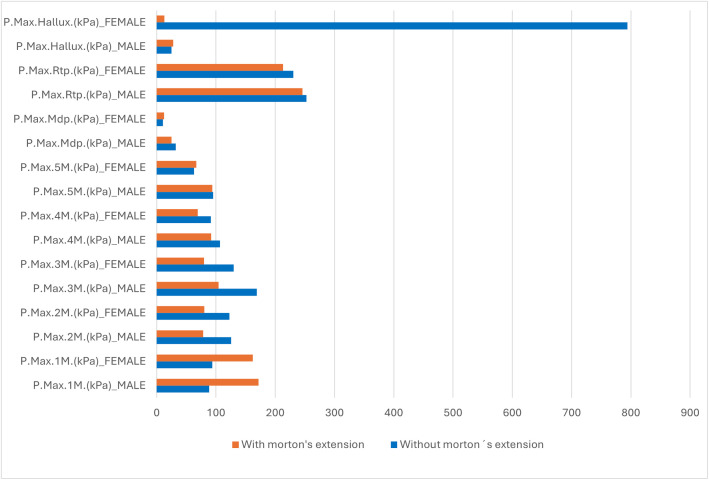
Comparative analysis of maximum plantar pressures in static conditions: impact of Morton’s Extension by sex. Legend of abbreviations: P.Max.1M (kPa), maximum pressure at the head of the first metatarsal; P.Max.2M (kPa), maximum pressure at the head of the second metatarsal; P.Max.3M (kPa), maximum pressure at the head of the third metatarsal; P.Max.4M (kPa), maximum pressure at the head of the fourth metatarsal; P.Max.5M (kPa), maximum pressure at the head of the fifth metatarsal; P.Max.Mdf (kPa), maximum pressure in the midfoot; P.Max.Rtp (kPa), maximum pressure in the rearfoot; P.Max.Hallux (kPa), maximum pressure in the hallux.

Fig 5 shows a comparative histogram of the maximum plantar pressures recorded in different regions of the foot under static conditions. The blue bars represent the data obtained without Morton’s extension, while the orange bars show the data obtained with the extension. Under static conditions without the extension, the maximum plantar pressures were generally higher in men than women. However, notable exceptions were observed for women in P.Max.1M (kPa), which exhibited a slight increase in pressure, and P.Max.Hallux (kPa), which was significantly higher than in men. Statistically significant differences (p < 0.05) were identified in P.Max.3M (kPa) and P.Max.Mdp (kPa). Under static conditions. Morton’s extension showed a tendency to reduce maximum plantar pressures in various regions of the foot for both men and women except for P.Max.1M (kPa), which displayed an increase in both sexes.

In the variables where pressure reduction was observed in both sexes, the decrease was more pronounced in men for P.Max.2M (kPa) (Table 4) and Max.3M (kPa). Conversely, for P.Max.4M (kPa) and P.Max.Rtp (kPa), the pressure reduction was greater in women. In other regions of the foot, the effects of Morton’s extension varied by sex. For instance, P.Max.5M (kPa) and P.Max.Mdp (kPa) showed a decrease in men, while women showed an increase. In contrast, P.Max.Hallux (kPa) increased in men and decreased in women. Statistically significant differences (p < 0.05) were identified in P.Max.Mdp (kPa) and P.Max.Rtp (kPa).

**Fig 6:**
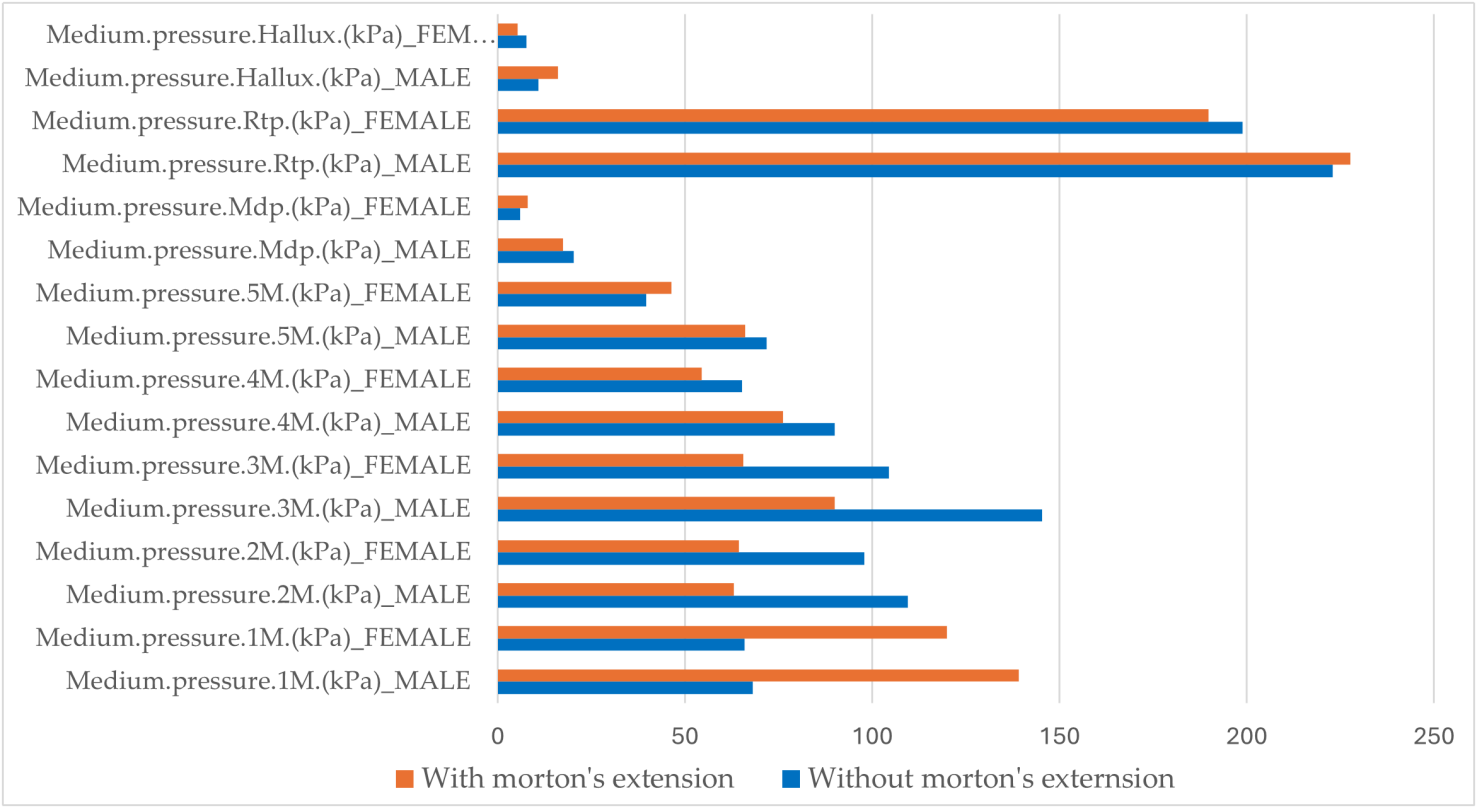
Comparative analysis of medium plantar pressures in static conditions: effect of Morton’s Extension by sex. Legend of abbreviations: Medium.pressure.1M (kPa), medium pressure at the head of the first metatarsal; Medium.pressure.2M (kPa), medium pressure at the head of the second metatarsal; Medium.pressure.3M (kPa), medium pressure at the head of the third metatarsal; Medium.pressure.4M (kPa), medium pressure at the head of the fourth metatarsal; Medium.pressure.5M (kPa), medium pressure at the head of the fifth metatarsal; Medium.pressure.Mdf (kPa), medium pressure in the midfoot; Medium.pressure.Rtp (kPa), medium pressure in the rearfoot; Medium.pressure.Hallux (kPa), and medium pressure in the hallux.

Fig 6 shows a comparative histogram of the medium plantar pressures recorded in different regions of the foot under static conditions. Under static conditions without Morton’s extension, the medium plantar pressures were higher in men for all the variables except for Mediu pressure.2M.(kPa), which was slightly lower in men. Statistically significant differences (p < 0.05) were observed in Medium pressure.3M (kPa), Medium pressure.4M (kPa), Medium pressure.5M (kPa), and Medium pressure.Mdf (kPa) (Table 5).

Under static conditions, Morton’s extension showed a tendency to reduce the medium plantar pressures in various regions of the foot for both men and women except for Medium pressure.1M (kPa), which increased in both sexes. Among the variables that showed a reduction in pressure in both sexes, the decrease was more pronounced in men for Medium pressure.2M (kPa), Medium pressure.3M (kPa), and Medium pressure.4M (kPa). In other regions of the foot, Morton’s extension produced sex-specific effects. For instance, Medium pressure.5M (kPa) and Medium pressure.Mdf (kPa) decreased in men but increased in women. Conversely, Medium pressure..Rtp (kPa) and Medium pressure.Hallux (kPa) increased in men and decreased in women. Statistically significant differences (p < 0.05) were identified in Medium pressure.3M (kPa), Medium pressure.4M (kPa), Medium pressure.Mdf (kPa), and Medium pressure.Rtp (kPa).

**Fig 7:**
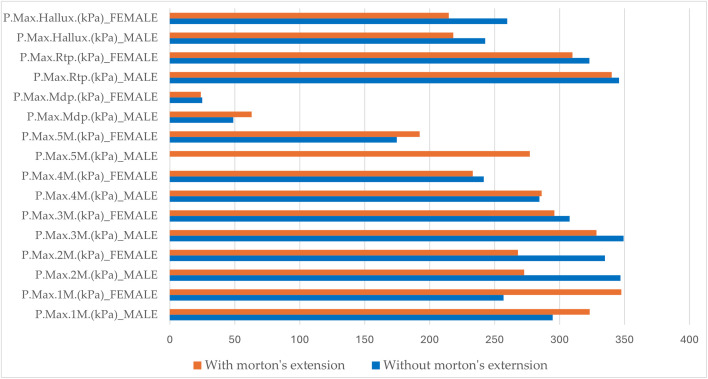
Comparative analysis of maximum plantar pressures in dynamic conditions: effect of Morton’s Extension by sex. Legend of abbreviations: P.Max.1M (kPa), maximum pressure at the head of the first metatarsal; P.Max.2M (kPa), maximum pressure at the head of the second metatarsal; P.Max.3M (kPa), maximum pressure at the head of the third metatarsal; P.Max.4M (kPa), maximum pressure at the head of the fourth metatarsal; P.Max.5M (kPa), maximum pressure at the head of the fifth metatarsal; P.Max.Mdf (kPa), maximum pressure in the midfoot; P.Max.Rtp (kPa), maximum pressure in the rearfoot; P.Max.Hallux (kPa), maximum pressure in the hallux.

Fig 7 shows a comparative histogram of the maximum plantar pressures recorded in different regions of the foot under dynamic conditions. Under dynamic conditions without Morton’s extension, the maximum plantar pressures were higher in men than women except for P.Max.Hallux (kPa), which was slightly higher among women. Statistically significant differences (p < 0.05) were observed in P.Max.3M (kPa), P.Max.Mdf (kPa), P.Max.Rft (kPa), and Contact.Surfaces (cm2) (Table 5). Under dynamic conditions, Morton’s extension demonstrated a tendency to reduce the maximum plantar pressures in various regions of the foot for both men and women except for P.Max.1M (kPa), which increased in both sexes. Among variables that decreased in both sexes, the decrease was more pronounced in men for P.Max.2M (kPa) and P.Max.3M (kPa). Conversely, for P.Max.Rft (kPa) and P.Max.Hallux (kPa), the pressure reduction was greater in women.

In other regions of the foot, Morton’s extension produced sex-specific effects. For example, P.Max.5M (kPa) decreased in men and increased in women. In contrast, P.Max.4M (kPa) and P.Max.Mdp (kPa) increased in men and decreased in women. Statistically significant differences (p < 0.05) were identified in P.Max.4M (kPa), P.Max.5M (kPa), P.Max.Mdf (kPa), and P.Max.Rft (kPa).

**Fig 8:**
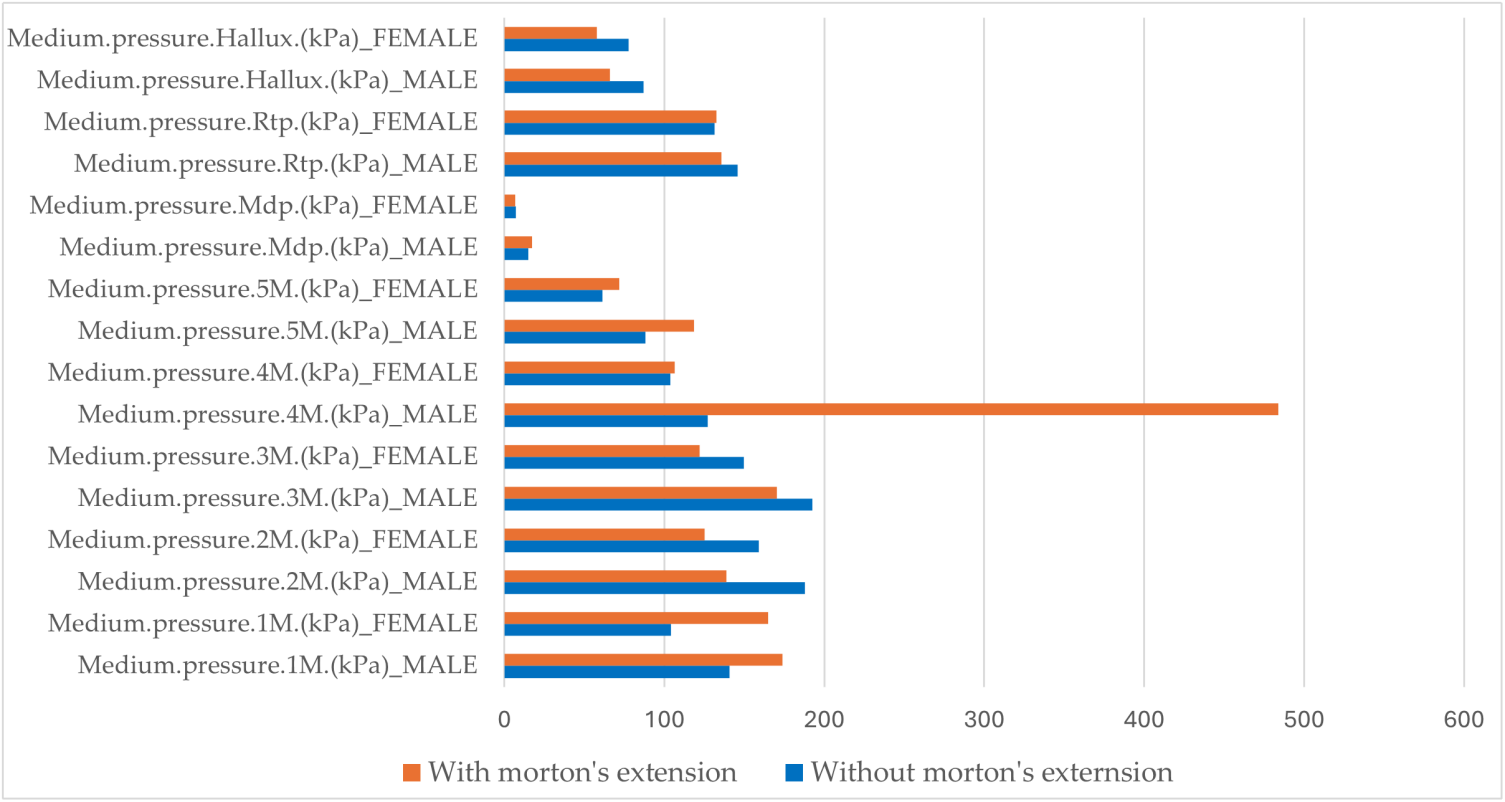
Comparative analysis of medium plantar pressures in dynamic conditions: effect of Morton’s Extension by sex. Legend of abbreviations: Medium.pressure.1M (kPa), medium pressure at the head of the first metatarsal; Medium.pressure.2M (kPa), medium pressure at the head of the second metatarsal; Mediu.pressure.3M (kPa), medium pressure at the head of the third metatarsal; Medium.pressure.4M (kPa), medium pressure at the head of the fourth metatarsal; Medium.pressure.5M (kPa), medium pressure at the head of the fifth metatarsal; Medium.pressure.Mdf (kPa), medium pressure in the midfoot; Medium.pressure.Rtp (kPa), medium pressure in the rearfoot; Medium.pressure.Hallux (kPa), and medium pressure in the hallux.

Fig 8 presents a comparative histogram of the medium plantar pressures recorded in different regions of the foot under dynamic conditions. Under dynamic conditions without Morton’s extension, the medium plantar pressures were higher in men than women. Statistically significant differences (p < 0.05) were observed in Medium pressure.1M (kPa), Medium pressure.2M (kPa), Medium pressure.3M (kPa), Medium pressure..5M (kPa), Medium pressure.Mdf (kPa), and Contact.Surfaces (cm2) (Table 5).

Under dynamic conditions, Morton’s extension tended to reduce the medium plantar pressures in various regions of the foot for both men and women except for Medium pressure.1M (kPa) and Medium pressure.4M (kPa), which increased in both sexes. Among variables that decreased for both sexes, the reduction was similar between men and women for Medium pressure.Hallux (kPa) and Medium pressure.3M (kPa). However, for Medium pressure.2M (kPa), the reduction was greater in men than women.

In other regions of the foot, Morton’s extension produced sex-specific effects. For instance, Medium pressure.Rft (kPa) decreased in men and increased in women. In contrast, Medium pressure.Mdf (kPa) increased in men and decreased in women. Statistically significant differences (p < 0.05) were identified in Medium pressure.3M (kPa), Medium pressure.5M (kPa), Medium pressure.Mdf (kPa), and Contact.Surfaces (cm^2^) (see Table 5)

## 4. Discussion

This study analyzes the impact of the application of Morton’s extension on variations in plantar pressure distribution between men and women. The main finding was that this orthotic element produces sex-dependent effects under both static and dynamic conditions, eliciting distinct biomechanical responses. In the absence of Morton’s extension, males exhibited significantly higher plantar pressures across most foot regions compared to females however, this trend was reversed in specific areas.

Morton’s extension revealed sex-specific effects on plantar pressure distribution. In males, it resulted in a marked reduction in pressure beneath the lateral metatarsal heads and the midfoot, indicating effective load redistribution. In females, although dynamic pressure under the fourth metatarsal decreased, mean pressures increased at the fifth metatarsal and midfoot. In a study conducted by Sims et al., an increase in pressure in the lateral portion of the midfoot was observed in men compared to women, considered a potential risk factor for the development of fifth metatarsal stress fractures [56]. This type of fracture is not uncommon in the athletic population, as it has been reported to account for up to 10% of injuries in female athletes and 22% in female track and field athletes [57]. These injuries are often a consequence of abnormal bone remodeling due to repeated exposure to stress or continuous mechanical impacts [58] which may necessitate temporary or even permanent withdrawal from sports participation. Furthermore, factors predisposing to stress fractures are also associated with an increased risk of osteoporosis [59], a bone disease affecting approximately 6.3% of men and 21.2% of women over 50 years of age [60] This is particularly relevant in the present study, as the application of Morton’s extension induced an increase in mean pressure in the fifth metatarsal and midfoot, potentially heightening the risk of overload-related injuries.

In the rearfoot, the orthotic produced a more substantial reduction in plantar pressure in females under both static and dynamic conditions, whereas males experienced a slight increase during static stance. Elevated plantar pressure in the rearfoot is an intrinsic factor associated with heel pain secondary to plantar fasciosis, which accounts for approximately 11% to 25% of cases. This condition has been linked to a decrease in quality of life and shows a higher prevalence in women aged between 40 and 60 years [61]. The observed modifications in plantar pressure may be explained by greater ligamentous laxity and lower arch stiffness in women [62], which are associated with relatively higher serum levels of estradiol and progesterone hormones shown to increase ligament laxity and reduce foot rigidity [62,63]. This hormonally induced laxity can influence both the structural architecture and functional behavior of the foot, predisposing to alterations in arch alignment that, depending on individual biomechanical characteristics, may manifest as excessive pronation (flatfoot) or accentuated supination (high arch) [64,65]. In contrast, men typically present greater stiffness in the plantar arch [66], resulting in a reduced adaptive capacity in foot positioning. Furthermore, several studies have demonstrated that arch height fluctuates throughout the menstrual cycle [67], reinforcing the role of hormonal factors in sex-related biomechanical differences. Within this context, our findings suggest that Morton’s extension exerts a more pronounced effect in female feet, inducing a greater varus alignment and a corresponding reduction in the contact area within this región [68]. According to Grady et al., plantar elements placed beneath the first ray may increase external rotation and inversion of the rearfoot. Other authors have examined the influence of forefoot wedges on pronation-supination movement at the subtalar joint to identify correlations between these biomechanical alterations and induced changes in adjacent structures [69, 70]. Based on these results, Morton’s extension could represent a valuable tool for improving rearfoot pressure distribution, particularly in the management of heel pain.

Under static conditions, women showed higher pressure values beneath the first metatarsal head and the hallux, which may be linked to their greater predisposition to forefoot disorders such as hallux limitus [24]. Consistent with our findings, previous research has demonstrated that this condition leads to a significant increase in peak pressure beneath the hallux and the first metatarsal head compared with healthy subjects [25,26]. Furthermore, other studies have reported that women exhibit significantly higher peak pressures not only under the hallux but also under the lesser toes, the forefoot, and the medial foot region, both in standing and during gait [19], reinforcing the presence of a more pronounced plantar overload pattern in the female foot.

Evidence suggests that sex differences are not solely attributable to anthropometric factors such as body weight or height, but rather vary according to the variable analyzed and do not follow a uniform pattern [51]. In a longitudinal study, Pau et al. [71] found that excess body mass increased plantar pressure in the midfoot and forefoot similarly in both sexes. However, even among individuals with normal body weight, women exhibited higher plantar pressures and larger contact areas. Studies examining the use of school backpacks in overweight or obese children have also shown that girls experience a more generalized increase in plantar pressure, particularly in the forefoot region [72]. These findings confirm the existence of sex-based differences that cannot be explained solely by anthropometric variations. Earlier research focused primarily on foot dimensions, concluding that the female foot represented a scaled-down version of the male foot, with shorter length and narrower width [18]. Nevertheless, plantar pressure variables do not behave uniformly or act merely as moderators, suggesting a complex interaction between biomechanical and physiological factors [73].

The application of Morton’s extension resulted in a reduction of plantar pressures in both sexes. It is well established that elevated plantar pressure can lead to hyperkeratosis, pain, and discomfort in the forefoot región [74]. Several factors may explain why this area experiences higher stress levels compared to the rearfoot; notably, the thickness of the soft tissues is approximately 36% to 48% greater in the rearfoot than in the forefoot [75] which reduces the latter’s capacity to absorb repetitive loads. Moreover, it is relevant to consider the clinical plantar pressure threshold of 207 kPa proposed by Owings et al. [33], which defines the risk limit for plantar ulceration in diabetic patients. In this context, Morton’s extension may represent an effective therapeutic alternative to redistribute plantar load and decrease the risk of injury in populations highly susceptible to overpressure.

These findings reveal an implicit difference in the magnitude of the effect between men and women, highlighting the need to establish specific dosimetry and customized orthotic heights tailored to the individual characteristics of each patient. Such personalization could optimize the therapeutic efficacy of biomechanical interventions and improve clinical outcomes according to the morphological and functional profile of each user.

In this study, a statistical approach based on mixed models was employed, allowing for an integrated analysis of the interaction between individual factors and experimental conditions and implicit sex-related differences both before and after the application of Morton’s extension. The use of mixed-model analysis in our research allowed for a more precise characterization of intra- and interindividual variability, revealing subtle sex-related differences prior to the intervention, significant differences were observed under static conditions in the mean pressure of the rearfoot and, under dynamic conditions, in both the maximum and mean pressures beneath the first metatarsal head. Moreover, during dynamic testing, notable differences were found in contact area and step duration, in contrast to the findings of Murphy et al.[29], who reported no sex-related variations in midfoot plantar pressure. It can be inferred that, even in the absence of intervention, dynamic conditions elicit greater sex-related differences, likely due to higher biomechanical loads and pressures inducing distinct gait behaviors. We posit that these differences particularly affect the midfoot region, where ligamentous elasticity is crucial for biomechanical function. It is plausible that women exhibit greater movements in the midtarsal and Lisfranc joints, a phenomenon that has not yet been directly investigated, but which is consistent with studies reporting a higher prevalence of flatfoot in the female population [76].

Unlike previous studies, the use of mixed-model analysis in our research allowed for a more precise characterization of intra- and interindividual variability, revealing subtle sex-related differences [77]. Following the application of Morton’s extension, statistically significant differences were identified in the maximum pressure beneath the first metatarsal and in the mean pressure of the rearfoot. Additionally, under dynamic conditions, the contact area also exhibited significant sex-related variations, reinforcing the influence of sex as a determining factor in the biomechanical response of the foot to this intervention. We consider that the greater contact area observed in women may be associated with increased soft tissue extensibility [62] and with the higher predisposition to flatfoot described in the female population [21]. In contrast, cavus feet are characterized by a reduction in total contact surface and in the load beneath the first toe [78].

The authors acknowledge several limitations that should be addressed in future research. Although some authors have suggested that controlling gait velocity may enhance the reliability of plantar pressure measurements [79], the most appropriate approach for doing so, especially considering sex-related differences, remains unclear.Increasing the sample size with a balanced sex distribution and including a non-intervention control group would further strengthen the robustness of the findings. Also: a proportion of individuals with joint hypermobility lacked a formal clinical diagnosis, which may have influenced the biomechanical interpretation of plantar pressure distribution. This limitation underscores the importance of integrating clinical assessments with experimental measurements in future studies. Finally, the absence of advanced mechanistic analyses represents an additional shortcoming. Although the experimental data revealed sex-related differences in plantar pressure distribution, these hypotheses were not validated through dynamic musculoskeletal simulations or finite element stress analysis. Incorporating such computational approaches could yield more comprehensive insights, as they have proven valuable in other contexts, including the optimization of lower-limb movement strategies [80] and gait analysis following fifth metatarsal fractures [81].

Plantar pressure parameters represent a valuable clinical tool for guiding the most appropriate therapeutic strategies and for preventing complications that can significantly affect a patient’s quality of life [82]. In this context, orthotic personalization based on sex-related biomechanical differences becomes critically important, as it enables the optimization of treatment efficacy, improvement of functional performance, and reduction of fatigue, pain, and injuries resulting from imbalanced plantar load distribution. For instance, the use of a Morton’s extension with a lower height in women, combined with an individualized clinical assessment to determine the optimal height for each patient, could be an effective strategy for controlling pressure in the fifth metatarsal region. Moreover, its application as a therapeutic tool to reduce rearfoot overload in certain pathologies further underscores its clinical potential. Traditionally, Morton’s extension has been primarily employed for the management of hallux-related disorders; however, the results of this study substantially expand its potential indications and highlight the importance of considering sex-related differences intrinsic to foot structure and biomechanics in the design and prescription of personalized orthopedic interventions.

Further studies must be conducted to verify with a large sample with a sex static analysis as a factor to continue the progression of orthotic treatments.

## 5. Conclusions

The results of this study demonstrate that there are sex differences in foot, and is the reason that the Morton’s extension produces differentiated effects on plantar pressure distribution between men and women, both under static and dynamic conditions.

Significant differences were observed in the maximum pressure under the first metatarsal head and in the mean pressure at the rearfoot after the application of the Morton’s extension, as well as in dynamic variables such as contact area and step duration.

Sex differences extis in Morton Extension orthotic application, and sex seems to be a determining factor in the foot’s response to orthotic modifications, reinforcing the need for a personalized prescription approach. Adjusting the height and geometry of the Morton’s extension according to each patient’s morphological and functional characteristics including sex.

Morton’s extension may serve as an effective tool not only for managing hallux-related pathologies but also for preventing and treating heel pain and other disorders associated with rearfoot overload. The sex-specific customization of orthotic treatments, particularly in women, could optimize plantar load redistribution and reduce the incidence of chronic pathologies, leading to lower healthcare costs and a decreased need for interventional treatments or surgical procedures in advanced stages.
